# Hale’s Epitome of Anatomy

**Published:** 1904-02

**Authors:** 


### Bibliography.

Hale's Epitome of Anatomy. A Manual for Students and Physicians. By Henry E. Hale, A.M.., M.D., Assistant Demonstrator of Anatomy College of Physicians and Surgeons (Columbia University) New York. In one 12mo volume of three hundred and eighty-four pages, with seventy-one illustrations. Lea Brothers & Co., Publishers, Philadelphia and New York, 1903.

This volume covers three hundred and eighty-four pages and seventy-one engravings, yet the matter is so carefully condensed that, like the others of the Lea series, it can be carried in the pocket for ready reference.

Every medical and dental student regards his Gray with peculiar reverence, but this large volume, wonderful in its simplicity and teaching force, cannot readily be handled at all times, and the student needs just such an epitome for his daily work. The foundation study of anatomy must necessarily be the same, whether it be derived from larger or smaller work, but there are manuals and manuals, each one aiming to give the subject in condensed form, but this one, like the others of the series, shows a marked ability in condensing without loss by abbreviation. The subjects follow each other in orderly sequence, and subjoined to these are a series of questions. These may, possibly, be of more value here than in the other books of the series, in that they enable the student to give a self-examination at the critical periods of college and State examinations. The condensed thoroughness of this book makes it, as well as the others, of very great value.

